# Comparative Analysis between Short Stem and Conventional Femoral Stem in Patients with Osteonecrosis of Femoral Head: Metha Stem and Excia Stem

**DOI:** 10.1111/os.12684

**Published:** 2020-05-29

**Authors:** Sung Soo Kim, Hyeon Jun Kim, Ki Woong Kim, Young Hun Jung, Si Young Heo

**Affiliations:** ^1^ Department of Orthopaedic Surgery College of Medicine, Dong‐A University Busan, South Korea; ^2^ Department of Orthopaedic Surgery Dong Kang Hospital Ulsan South Korea

**Keywords:** Conventional femoral stem, Osteonecrosis, Short stem, Total hip arthroplasty

## Abstract

**Objective:**

To compare the intraoperative, radiological, and clinical short‐term outcomes of cementless total hip arthroplasties (THA) using a short stem (SS) and a conventional femoral stem (CS) in a randomized prospective control study.

**Methods:**

From June 2011 to October 2017, patients who underwent cementless THA for idiopathic osteonecrosis of the femoral head were recruited. Patients had a minimum 2 years of follow‐up after the operation. The patients were divided into two groups: those who underwent THA using an SS and those who underwent THA using a CS. SS were used in 34 patients (41 hips) and CS were used in 41 patients (45 hips). In both groups, the same cup was used in all cases, and the mean follow‐up periods were 63 (26–101) months in the SS and 64 (26–101) months in the CS groups. Intraoperative, clinical, and radiological evaluations were performed for the two groups.

**Results:**

There was no difference in the demographics of the two groups. There was one patient with a proximal femoral crack in the SS group and one with a distal femoral crack in the CS group. Clinically, the mean Harris hip score was improved in both groups at 2‐year follow‐up. Radiographically endosteal osseointegrations were found in 40 of 41 cases in the SS group and in 44 of 45 cases in the CS group. There was one case of dislocation in each group. In the SS group, the acetabular cup was changed and repositioned 7 months after the initial operation. Stem loosening, infection, ceramic breakage, and varus/valgus change were not observed. There was a statistically significant lower stress shielding effect in the SS group. There were no differences in vertical/parallel offset and leg length discrepancy.

**Conclusion:**

The intraoperative, radiological, and clinical evaluations in both groups showed good outcomes and there was no statistically significant difference between the two groups.

## Introduction

The hip is one of the major weight‐bearing joints in the body. Osteonecrosis of the femoral head (ONFH) is one of the primary causes of hip pain in younger adults and typically progresses to collapse of the femoral head and secondary osteoarthritis of the hip.^1^ Total hip arthroplasty (THA) is the most common surgical approach used in patients with ONFH. Along with the advances in materials engineering, osseointegration has been progressively improving and the use of cementless artificial hip joint has been gradually increasing.^2^, ^3^, ^4^, ^5^ As the prevalence of cementless THA has increased, surface treatment methods that promote osseointegration of the femoral stem, the acetabular cup, and bearing surfaces have been steadily advanced to improve in clinical results and survival rates.^6^, ^7^, ^8^, ^9^ Over the past decade, short femoral stems have drawn increasing attention and the many advantages of short stems have been advocated, including preservation of the femoral bone stock, optimization of proximal load transfer, the minimally invasive surgical procedures and less injury to muscles around the greater trochanter, and easier surgical procedures for revision.^10^, ^11^, ^12^ The authors of the present study examined the difference in the use of a short stem (SS) and a conventional femoral stem (CS) when performing THA in patients with ONFH. We conducted a prospective randomized comparative study to identify differences in radiological and clinical outcomes between the two different stems.

## Materials and Methods

### *Subjects*

**Figure 1:**
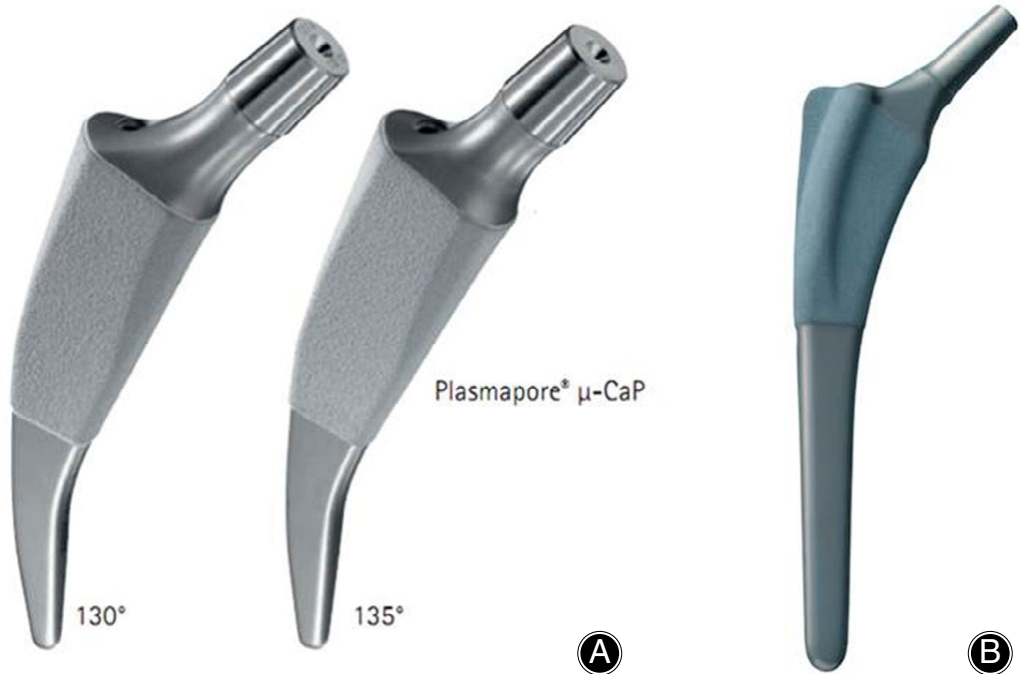
Photograph of short hip stem (A) Metha and (B) Excia prostheses.

This study was performed after gaining institutional review board approval from the authors' institute. From June 2011 to October 2017, a randomized prospective study was performed in patients who underwent cementless THA after being diagnosed as having femoral head osteonecrosis. Patients were randomly assigned to either the SS group or the CS group. When performing cementless THA, the SS used was the Metha short hip stem system (Aesculap, B. Braun, Germany) and the standard length stem used was the Excia hip stem system (Aesculap, B. Braun, Germany) (Fig. 1).

Inclusion criteria were patients who were diagnosed with femoral head osteonecrosis and who were randomly selected for application of cementless THA with Metha or Excia stems. Of these, patients who could be followed up for a minimum of 2 years were included in the analysis. Exclusion criteria were patients whose femoral stems were converted to cemented stems during the operation. Patients were excluded from the analysis if they were not available for 2‐year follow‐up. Age was not an inclusion or exclusion factor.

Consequently, there were a total of 34 patients (41 hips) in the Metha stem group and a total of 41 patients (45 hips) in the Excia stem group.

### *Surgical Methods and Postoperative Management*

All operations were performed by a single surgeon. Under general anesthesia, patients were placed in the lateral decubitus position. The modified Gibson posterolateral approach was used, and fixations were the non‐cemented type. The Metha short hip stem system used in the SS group has a tapered wedge design and was fixed into the cortical bone of the neck, cut higher than the usual level of the femoral neck cut. The Excia hip stem system used in the CS group was fixed in the metaphysis by press‐fitting the stem to the neck cut level typically made. Both stems are designed to promote bone ingrowth using dicalcium phosphate coating together with surface treatment involving coating with microporous pure titanium in the proximal region (Fig. 1). The Plasmacup acetabular cup system (Aesculap AG, Germany) was used in all patients in both groups, and Biolox delta (Aesculap AG, Germany) was used for the bearing surface. The acetabular cup and implants were fixed cementless with press‐fit, and if necessary, two or three cancellous screws were used to fix the acetabular cup. Muscle strengthening exercises and partial weight‐bearing were permitted immediately after surgery. After starting partial weight‐bearing using a walker or crutches, progression to full weight‐bearing was gradually commenced.

### *Intraoperative Evaluation*

Intraoperative femoral fractures during stem insertion were graded using the Mallory classification system,^13^ and duration of operation and blood loss were assessed.

### *Clinical Evaluation*

Harris hip scores (HHS)^14^ and the Oswestry disability index (ODI)^15^ measured preoperatively and at 2‐year follow‐up were compared to evaluate the functional outcomes of the hip. HHS of 90 points or above were defined as excellent, 80–90 points as good, 70–80 points as fair, and less than 70 points as poor. For the ODI, hips with a score of 0%–20% are considered as having mild dysfunction, 21%–40% as moderate dysfunction, 41%–60% as severe dysfunction, and 61%–80% is considered disabling. For cases with a score of 81%–100%, the patient is either bedridden long term or is exaggerating the impact of pain on their life. In addition, the presence or absence of postoperative complications was determined, including hip dislocation and surgical site infection, thigh pain at final follow‐up, claudication, clicking or squeaking sound, and periprosthetic fractures.

### *Radiological Evaluation*

**Figure 2:**
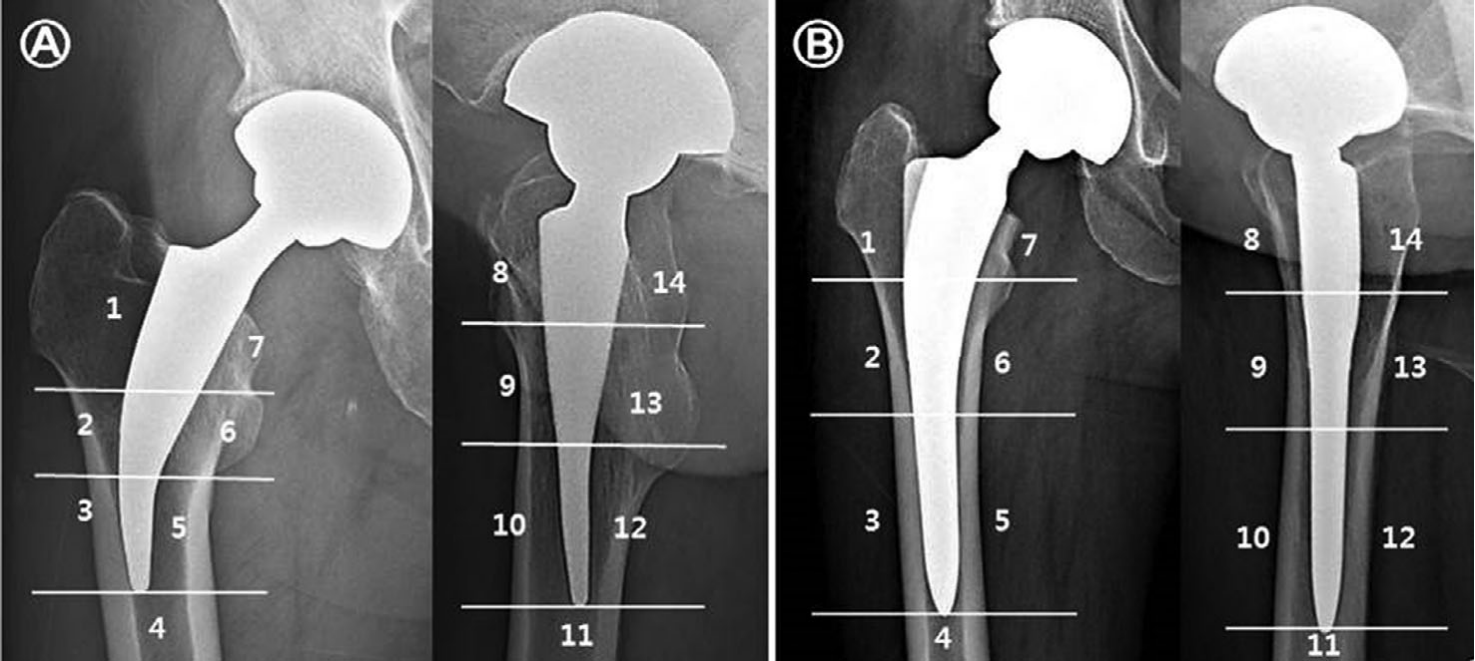
Definition of Gruen's periprosthetic zones. (A) Short hip stem Metha. (B) Excia prosthesis.

**Figure 3:**
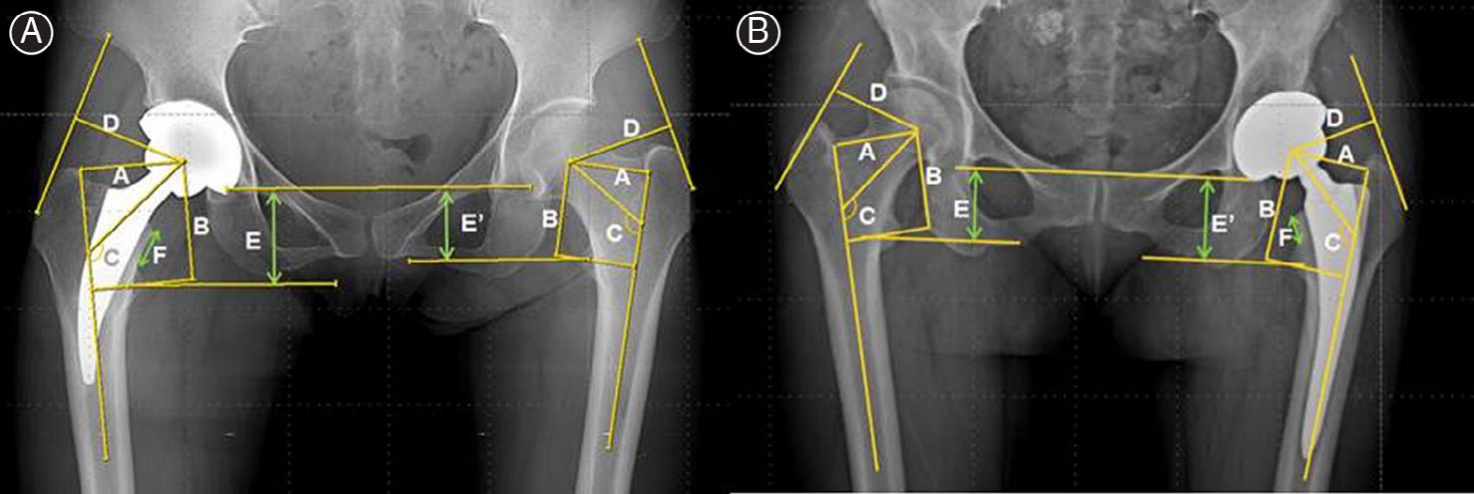
Radiologic measurement method. (A) Short hip stem Metha. (B) Excia prosthesis. A: Femoral offset, B: Vertical offset, C: Centrum–collum–diaphyseal (CCD) angle, D: Abductor moment arm, E–E′: Leg length discrepancy, F: Neck cutting level.

All patients were followed‐up preoperatively and postoperatively, at the 6th week, the 3rd and 6th month, and the 1st year, and at annual intervals thereafter. Follow‐up radiographs were compared to previous imaging to assess radiographic changes. Radiographs were examined by dividing the proximal femur into seven Gruen zones^16^ to evaluate bony ingrowth, aseptic loosening, osteolysis, and other bone changes around the femoral stem (Fig. 2). Direct contact between the femoral component and the cancellous bone in Gruen zones was defined as osseointegration, and vertical subsidence of the femoral component by more than 3 mm or a varus/valgus misalignment greater than 5° was considered aseptic loosening.^17^, ^18^ At the last follow‐up, stress shielding around the two different stems was compared using Engh and Bobyn's criteria,^19^ and osteolytic lesions were examined according to the classification described by Zicat *et al*.^20^ Heterotopic ossification was determined using the classifications of Brooker *et al*.^21^. To determine the changes in femoral offset, preoperative and postoperative vertical/horizontal femoral offset and neck–shaft angle, leg length discrepancy (LLD) and femoral neck cut height were measured (Fig. 3). In addition, the presence of complications was determined, such as femoral cortical thickening and ceramic wear and breakage.

### *Statistical Analysis*

Statistical significance was determined by comparing demographic differences, intraoperative evaluation criteria, and clinical and radiological evaluation criteria between the two groups using the Student *t*‐test and the χ^2^‐test. All statistical analyses were performed using IBM SPSS Statistics 25 software (IBM, Armonk, NY, USA). *P*‐values of less than 0.05 were considered statistically significant.

## Results

There were no differences in patients' demographic characteristics between the two groups, including surgical site, sex, age, body mass index, and follow‐up period (Table 1).

**Table 1:** The demographics of the two stem groups

Parameters	Type of implant		*P*‐value
	Short stem (Metha)	Conventional stem (Excia)	
Number of patients (*n*)	34	41	NA
Number of hips (*n*)	41	45	NA
Side (right : left)	22:19	24:21	0.967
Sex (male : female)	18:16	21:20	0.599
Mean age (years) (range, SD)	52.4 (21–69, 12.3)	52.2 (21–68, 11.8)	0.952
Mean body mass index (kg/m^2^) (range, SD)	22.7 (17.8–27.3, 2.5)	23.6 (16.9–32.9, 4.1)	0.337
Follow‐up (months)	63 (26–101)	64 (26–101)	0.415

NA, not applicable

### *Intraoperative Results*

**Figure 4:**
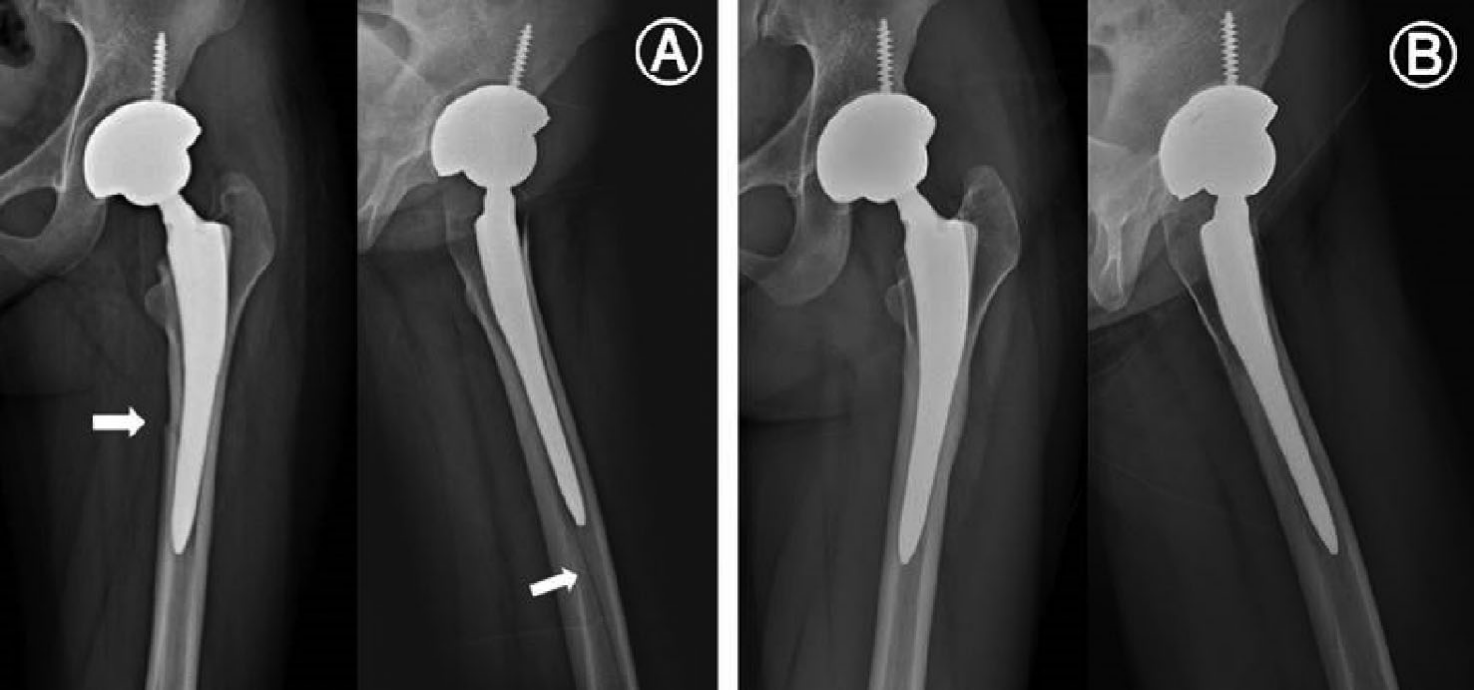
(A) Intraoperative femoral fracture, extending to the femur shaft (Mallory classification type C) occurred in 1 case of Excia prosthesis. (Arrow) (B) The fracture was treated by nonoperative management and resulted in bone union at 8 months.

A Mallory type I fracture in the proximal femur occurred intraoperatively in one case (2.4%) in the Metha stem group and a Mallory type III fracture, extending to the femoral shaft, occurred in one case (2.2%) in the Excia stem group during stem insertion (Fig. 4). These 2 cases were conservatively managed with no additional surgical procedures (such as cerclage wiring), and fracture union was confirmed at follow‐up. No significant difference was found in operative time and blood loss between the two groups (Table 2).

**Table 2:** Intraoperative results of the two stem groups

Parameters	Type of implant		*P*‐value
	Short stem (*n* = 41)	Conventional stem (*n* = 45)	
Average operative time (min)	119 (69–170)	110 (75–145)	0.142
Average estimated blood loss (mL)	518 (200–1000)	594 (400–1200)	0.120
Intraoperative periprosthetic fracture (*n*, %)			NA
Type I	1 (2.4)	NA	
Type II	NA	NA	
Type III	NA	1 (2.2)	

NA, not applicable

### *Clinical Results*

**Figure 5:**
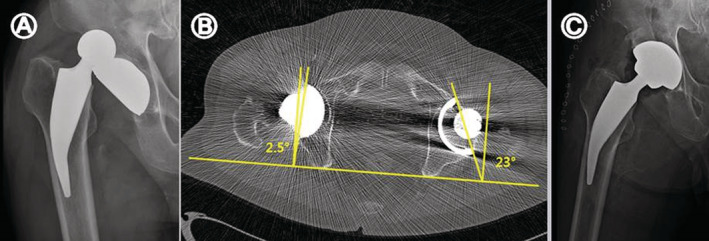
(A) Radiograph of a 66‐year‐old man with recurrent dislocation of implant at 2 weeks postoperatively. (B) Axial CT scan image demonstrating the decreased acetabular component anteversion of right hip (2.5°). (C) Revisional replacement of acetabular cup was done 7 months after the primary surgery.

The mean HHS improved from 62.5 (31–74) preoperatively to 94.2 (84–98) at the final follow‐up in the Metha stem group, and from 60.7 (28–76) preoperatively to 93.8 (79–98) at the final follow up in the Excia stem group. The mean ODI improved from 32.5% (27%–39%) preoperatively to 16.2% (12%–20%) at the final follow up in the Metha stem group, and from 32.7% (28%–40%) preoperatively to 16.4% (14%–23%) at the final follow up in the Excia stem group. Two patients (4.4%) complained of thigh pain in the CS group, but they required no additional treatment. In both stem groups, there was one case (2.4%, 2.2%) of recurrent dislocation that appeared to be caused by inadequate anteversion of the acetabular component. Acetabular cup revision and realignment were performed in the Metha stem group. An abduction brace was used in the patient of the Excia stem group (Fig. 5). Periprosthetic fracture or infection did not occur, but squeaking from the surgical site occurred in one case in each group (Table 3).

**Table 3:** Clinical results at the final follow up

Parameters	Type of implant		*P*‐value	
	Short stem (*n* = 41)	Conventional stem (*n* = 45)		
Mean HHS (SD, range)	94.2 (7.9, 84 to 98)	93.8 (8.7, 79 to 98)	0.681
Mean ODI (SD, range)	16.2	16.4	0.210
Thigh pain (*n*, %)	0 (0)	2 (4.4)	0.135
Dislocation (*n*, %)	1 (2.4)	1 (2.2)	0.957
Clicking or squeaking sound (*n*, %)	1 (2.4)	1 (2.2)	0.957
Claudication (*n*, %)	0 (0)	0 (0)	NA
Infection (*n*, %)	0 (0)	0 (0)	NA
Postoperative periprosthetic fracture (*n*, %)	0 (0)	0 (0)	NA

HHS, Harris hip score; NA, not applicable; ODI, Oswestry disability index; SD, standard deviation

### *Radiological Results*

**Figure 6:**
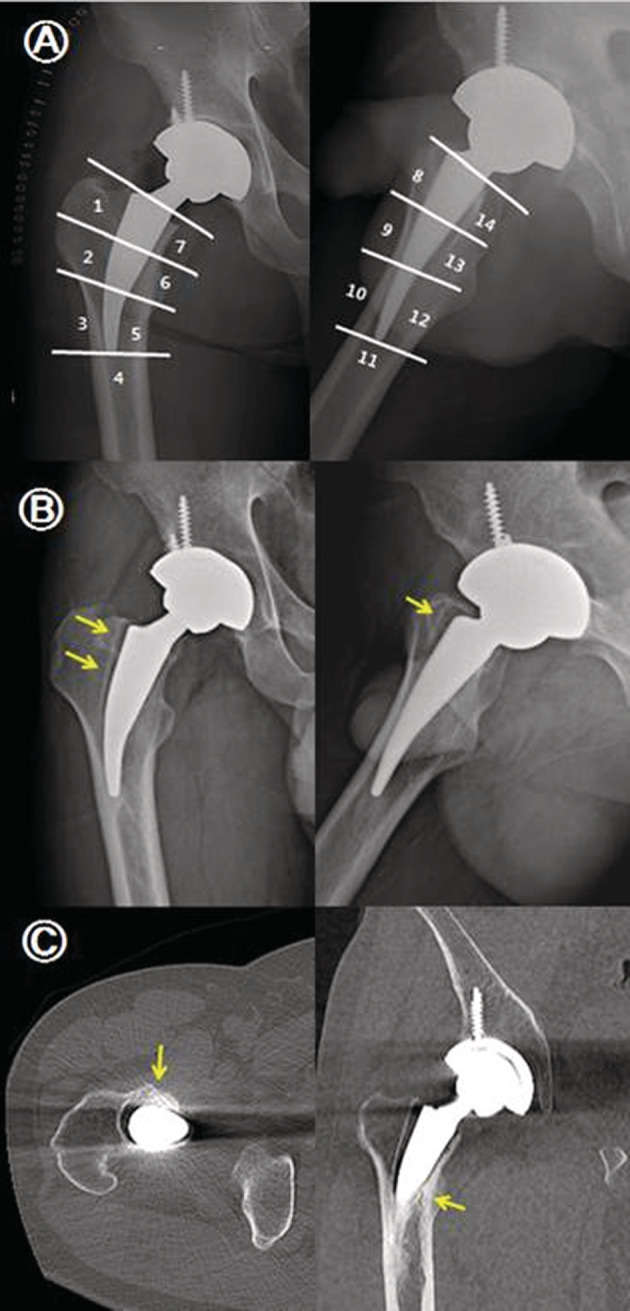
(A) Immediate postoperative radiograph of a 38‐year‐old man with avascular necrosis of the femoral head and definition of Gruen's periprosthetic zones. (B) Radiographs at 42 months postoperatively with pedestal formation at the distal smooth portion radiolucent line at zones 1, 2, and 8. (C) Axial and coronary CT scan images showing progression of osseointegration at sites other than osteolysis (arrow).

No significant difference was noted between the two groups in radiological evaluation criteria, excluding stress shielding of the proximal femur (Table 4). The neck cut level was significantly higher in the Metha stem group, but the final LLD after surgery showed no statistically significant difference between the two groups. Bony ingrowth with good osseointegration was observed in 40 (97.6%) of 41 cases in the SS group and in 44 (97.8%) of 45 cases in the CS group. In the Gruen zones, osseointegration was most commonly seen in Gruen zone 6 in the Metha stem group and in Gruen zone 2 in the Excia stem group (Table 5). Osteolysis manifesting the radiolucent line around the femoral stem on anteroposterior and lateral radiographs was detected in one case in the SS group (Fig. 6). There were no cases with vertical subsidence more than 3 mm or of varus/valgus misalignment greater than 5° in either group. Grade 1 stress shielding in the proximal femur was shown in all cases in the Metha stem group. Grade 2 stress shielding was most commonly seen, in 37 cases (82.2%), and grade 1 or 3 stress shielding was seen in 4 cases (8.9%) each in the Excia stem group.

**Table 4:** Radiological results at the final follow‐up

Parameters	Type of implant		*P*‐value
	Short stem (*n* = 41)	Conventional stem (*n* = 45)	
Femoral stem position coronal plane (*n*, %)			‐
Neutral	41 (100)	45 (100)	
Varus	0 (0)	0 (0)	
Valgus	0 (0)	0 (0)	
Offset Analysis (SD, range)			
Difference of FO (mm)	+3.9 (2.7, −2.1–7.5)	+4.1 (3.1, −3.1–9.2)	0.793
Difference of VO (mm)	+5.4 (2.8, 1.5–13.9)	+6.2 (3.3, −1.1–13.4)	0.305
Difference of NSA (°)	+3.2 (3.5, −2.2–16.7)	+2.0 (1.7, −1.1–7.6)	0.128
Leg length discrepancy (mm)	6.9 (1.1–17.25)	7.3 (0.9–15.5)	0.75
Neck cutting level (mm)	18.7 (12.0–25.71)	14.0 (10.0–21.9)	0.00
Radiolucent line >2 mm (*n*, %)	1 (2.4)	0 (0)	0.331
Migration of femoral stem >3 mm (*n*, %)	0 (0)	0 (0)	NA
Cortical hypertrophy (*n*, %)	1 (2.4)	2 (4.4)	0.509
Stress shielding effect (*n*, %)			0.028
Grade I	41 (100)	4 (8.9)	
Grade II	0 (0)	37 (82.2)	
Grade III	0 (0)	4 (8.9)	
Grade IV	0 (0)	0 (0)	
Heterotrophic ossification (*n*, %)			0.367
Brooker's grade I	4 (9.8)	3 (6.7)	
Brooker's grade II	1 (2.2)	0 (0)	
Brooker's grade III	0 (0)	0 (0)	
Brooker's grade IV	0 (0)	0 (0)	

FO, femoral offset; NA, not applicable; NSA, neck shaft angle; VO, vertical offset.

**Table 5:** Radiographic osseointegration around the femoral stems by Gruen's classification

Zone by Gruen's classification	Short stem (*n* = 41) number (%)	Conventional stem (*n* = 45) number (%)	*P*‐value
1	7 (17.1)	7 (15.6)	0.385
2	12 (29.3)	28 (62.2)	0.121
3	37 (90.2)	3 (6.7)	0.014
4	NA	NA	NA
5	10 (24.4)	7 (15.6)	0.257
6	40 (97.6)	20 (44.4)	0.089
7	16(39.0)	9 (20.0)	0.093

NA, not applicable

Heterotopic ossification occurred in the abductor muscles in 4 of 41 hips (9.8%) and in the iliopsoas muscle in 1 hip (2.4%) in the SS group, while in the abductor region alone in 3 of 46 hips (6.7%) in the CS group. There was no case with ceramic wear and breakage in either group. Femoral cortical thickening was observed in one case in the Metha stem group and in 2 cases in the Excia stem group.

## Discussion

Over the past decade, minimally invasive surgery for THA has increasingly drawn attention and in this process, SS designs have also gained much interest. Short femoral stems have advantages over standard length stems by allowing the preservation of the femoral neck, a less invasive surgical approach, and easier revision surgery.^22^ Only a few studies have reported on the clinical outcomes of primary THA using SS prostheses with a long‐term follow up of more than 10 years. Huo *et al*.^23^ demonstrated that SS decreased thigh pain after surgery and obtained the same clinical and radiological results as CS. Wittenberg *et al*.^24^ reported that the cumulative 5‐year survival rate was 96.7% for the Metha SS used in the present study. With failure defined as stem revision due to stem‐related problems, the present study achieved excellent results, with a survival rate of 100% after a mean follow‐up of 63 and 64 months in SS and CS stem groups, respectively.

### *Design and characters of Metha and Excia Hip Stems*

The Metha and Excia hip stems used in this study were designed to promote bone ingrowth by obtaining initial stability with press‐fit and long‐term biological fixation with surface treatment in the proximal region. This stem design enables firm fixation as bone ingrowth mainly occurring in the proximal region minimizes proximal stress shielding. However, unlike the Excia stem, the Metha stem is anchored to the cortex of the femoral neck, with a high neck cut, and increases stability by achieving surface contact between the distal end of the stem and the posterolateral cortex.

### *Analysis of Intraoperative and Postoperative Results*

Local risk factors for intraoperative fractures during THA include press‐fit cementless hip prostheses, deformity of the proximal femur, revision surgery, and others. The force of the wedge effect imposed on the femur during insertion for cementless hip prostheses may cause proximal femoral fractures. The published literature reports that fracture rates during THA range between 1% and 6%.^25^ Huachen *et al*.^26^ suggested that the risk of intraoperative periprosthetic fractures was higher in CS because while it is likely that SS requires broaching prior to stem insertion, CS requires both reaming and broaching. Ryan *et al*.^27^ reported that intraoperative complications occurred at an incidence of 0.4% (1 of 269 cases) in SS, lower than the incidence of 3.1% (12 of 389 cases) when using the standard length stem. In the current study, the incidence of intraoperative complications was 2.4% (1 case) and 2.2% (1 case) in each group, with no significant difference. Complication rates were comparable to those of previous studies.

Although the exact cause of postoperative thigh pain has not yet been clarified, the possible causes are micromotion of femoral stems, local load transfer to the distal femur, and difference in strength between the prosthesis and bone. In our study, thigh pain was observed in 2 (4.4%) cases in the Excia stem, but there was no statistically significant difference. This outcome was comparable to the incidence described by Engh *et al*.^28^, where 8% of patients with standard length stems experienced pain.

Squeaking occurred in one case in each group. Lee *et al*.^29^ suggested that the higher inclination angle of the acetabular cups was a significant cause for squeaking in primary cementless THA using alumina bearings. The patients with squeaking had cup inclination angles greater than 60° on postoperative radiographs. Those 2 patients were simply observed because they had no pain or other clinical symptoms.

Postoperative recurrent dislocation occurred in 1 case in each group. Both patients had an increased risk of recurrent dislocation because they were frequent binge drinkers. A patient from the Metha stem group experienced eight recurrences of dislocation. In the radiographic assessment, the femoral head was found to be dislocated in the posterosuperior direction, which appeared to have resulted from insufficient acetabular inclination, by 2.5°. Without stem revision, this patient underwent revision by increasing the acetabular cup inclination using a larger acetabular component and recurrent dislocation was resolved completely. The other patient in the Excia stem group wore a hip abduction brace without additional surgery as the cup inclination was normal in the first dislocation, but the patient's compliance with treatment was low. Once the patient consistently followed clinical guidelines to prevent recurrences, dislocation no longer occurred.

In THA, the restoration of biomechanics such as offset and leg length is a critical aspect in achieving successful outcomes.^30^ This study compared changes in preoperative and postoperative offset between the two groups, with the femoral offset increasing in both groups. However, this increase in postoperative offset showed no statistical significance compared to preoperative offset, and no difference was found between the two groups.

The neck cut level was significantly higher in the SS group, but the final LLD after surgery demonstrated no significant difference between the two groups. There was no significant difference for vertical offset. We predicted that a higher neck cut would result in greater LLD and vertical offset, but LLD and offset were thought to be controlled by adjusting the neck cut level and stem length *via* preoperative templating. No previous studies have reported differences in LLD and offset using SS.

In THA, stress shielding primarily occurs in the proximal regions of bone ingrown implants. Bone loss caused by stress shielding can be indicated as one of the risk factors for loosening, but stress shielding alone does not fully describe the stress shielding effects from the implant. Salemyr *et al*.^31^ suggested that stress shielding effects were less in short hip stems compared to standard length stems. In line with this previous research, the SS group had (statistically significantly) less stress‐shielding effect compared to the CS group in the current study. Stem design affects load transfer, which influences stress shielding and bony ingrowth with osseointegration. SS prostheses were introduced to enhance the preservation of femoral bone stock. In the beginning, there were concerns about whether the use of SS would maintain the primary stability required for achieving osseointegration and stabilization, interfered by stem loosening and subsidence.^32^ This investigation aimed to explore osseointegration around the femoral component to ensure secondary stability by comparing two different stem designs. Osseointegration largely occurred in the proximally coated areas in both stems. When using SS, the Metha stem is anchored to the cortex of the femoral neck with a neck cut and its fixation is characterized by surface contact between the distal end of the stem and the lateral cortex. For this reason, osseointegration mainly occurred in Gruen zones 3 and 6 even without coating. Ludwig *et al*.^33^ states that a radiolucent line of more than 2 mm is evidence of stem loosening. In contrast, Kamada *et al*. report that the radiolucent line usually does not progress in the proximal femur.^34^ In our study, the radiolucent line in the proximal area did not affect the clinical results of patients.

### *Limitation and Aim of Study*

There are some limitations to note in this study. First, radiological assessment was conducted using simple X‐rays instead of 3D images obtained by 3D CT. Even though all anteroposterior and lateral view radiographs were used to overcome this limitation, the results of this study may be still limited. Second, anteroposterior and lateral X‐ray radiographs were taken with both hips internally rotated by 15°, but biased results may be obtained. Finally, the present study was limited by the relatively small sample size and the relatively short follow‐up period of 2 years. Therefore, long‐term follow‐up is warranted to further investigate complications.

The significance of this report is that various SS are currently used and reported, but there are few clinical reports on the Metha stem type. Therefore, we believe this is meaningful research despite the limitations mentioned above.

### *Conclusion*

This study performed THA in patients with ONFH using SS (the Metha stem) and CS (the Excia stem) with a mean follow‐up of 5 years, and clinical and radiological evaluations demonstrated good outcomes in both groups.
